# Allele-specific binding variants causing ChIP-seq peak height of histone modification are not enriched in expression QTL annotations

**DOI:** 10.1186/s12711-024-00916-4

**Published:** 2024-06-27

**Authors:** Mohammad Ghoreishifar, Amanda J. Chamberlain, Ruidong Xiang, Claire P. Prowse-Wilkins, Thomas J. Lopdell, Mathew D. Littlejohn, Jennie E. Pryce, Michael E. Goddard

**Affiliations:** 1 Agriculture Victoria Research, AgriBio Centre for AgriBioscience, Bundoora, VIC 3083 Australia; 2 School of Applied Systems Biology, La Trobe University, Bundoora, VIC 3083 Australia; 3 Faculty of Veterinary & Agricultural Science, University of Melbourne, Parkville, VIC 3010 Australia; 4 Research and Development, Livestock Improvement Corporation, Private Bag 3016, Hamilton, 3240 New Zealand

## Abstract

**Background:**

Genome sequence variants affecting complex traits (quantitative trait loci, QTL) are enriched in functional regions of the genome, such as those marked by certain histone modifications. These variants are believed to influence gene expression. However, due to the linkage disequilibrium among nearby variants, pinpointing the precise location of QTL is challenging. We aimed to identify allele-specific binding (ASB) QTL (asbQTL) that cause variation in the level of histone modification, as measured by the height of peaks assayed by ChIP-seq (chromatin immunoprecipitation sequencing). We identified DNA sequences that predict the difference between alleles in ChIP-seq peak height in H3K4me3 and H3K27ac histone modifications in the mammary glands of cows.

**Results:**

We used a gapped k-mer support vector machine, a novel best linear unbiased prediction model, and a multiple linear regression model that combines the other two approaches to predict variant impacts on peak height. For each method, a subset of 1000 sites with the highest magnitude of predicted ASB was considered as candidate asbQTL. The accuracy of this prediction was measured by the proportion where the predicted direction matched the observed direction. Prediction accuracy ranged between 0.59 and 0.74, suggesting that these 1000 sites are enriched for asbQTL. Using independent data, we investigated functional enrichment in the candidate asbQTL set and three control groups, including non-causal ASB sites, non-ASB variants under a peak, and SNPs (single nucleotide polymorphisms) not under a peak. For H3K4me3, a higher proportion of the candidate asbQTL were confirmed as ASB when compared to the non-causal ASB sites (*P* < 0.01). However, these candidate asbQTL did not enrich for the other annotations, including expression QTL (eQTL), allele-specific expression QTL (aseQTL) and sites conserved across mammals (*P* > 0.05).

**Conclusions:**

We identified putatively causal sites for asbQTL using the DNA sequence surrounding these sites. Our results suggest that many sites influencing histone modifications may not directly affect gene expression. However, it is important to acknowledge that distinguishing between putative causal ASB sites and other non-causal ASB sites in high linkage disequilibrium with the causal sites regarding their impact on gene expression may be challenging due to limitations in statistical power.

**Supplementary Information:**

The online version contains supplementary material available at 10.1186/s12711-024-00916-4.

## Background

Sequence variants affecting complex traits (here called quantitative trait loci or QTL, meaning the causal variant) are enriched in functional regions of the genome such as transcription factor (TF) binding sites, promoters, and enhancers [1, 2]. One way a QTL or functional variant could influence phenotypes of a complex trait is by regulating gene expression (i.e., expression QTL; eQTL) [3]. *cis* eQTL are regulatory variants affecting gene expression that are located nearby the gene they regulate on the same chromosome, and they may be identified by exploring the association between the variation in the level of gene expression and the nearby sequence variants. However, due to linkage disequilibrium (LD) with numerous nearby variants, it remains challenging to pinpoint them [4, 5].

ChIP-seq (chromatin immunoprecipitation followed by sequencing) is a technique that enables the identification of functional genomic regions e.g., histone modifications in a specific tissue or cell-type. The technique involves utilizing antibodies to capture the DNA or genome regions marked by histone modifications, isolating, and subsequently sequencing them [6]. When the resulting DNA sequences are aligned to the genome, they form peaks that serve as a feature. This feature (i.e., height of the peak) is indicative of the level of histone modification. According to [7], variants that are putatively causal for differences in ChIP-seq peak height are typically found within the peaks whose height they affect. The same authors also found that eQTL were enriched as histone modification QTL (hQTL), thus providing evidence that non-coding functional regions regulate gene expression [7]. Therefore, identifying sequence variants affecting histone modification peak height can potentially aid in the discovery of *cis* eQTL. If an individual is heterozygous at an hQTL that operates in *cis*, it will cause a difference between the homologous chromosomes in the ChIP-seq peak height. Another way to describe this is that the ratio of the two alleles in the ChIP-seq reads mapped to this position will differ from 1:1. This phenomenon is referred to as allele-specific binding (ASB) [7, 8], and the heterozygous site putatively causing this event as an ASB QTL (asbQTL). There is also a possibility that a heterozygous site under a peak will show ASB because it is in LD with a causal site, in which case it is here called non-causal ASB.

Identifying causal variants within regulatory regions continues to pose a significant challenge, primarily due to the presence of LD, which complicates the differentiation of a true causal variant from other variants within the same regulatory region. However, if an hQTL can be predicted from the underlying DNA sequence, this removes the ambiguity caused by LD. To this aim, several sequence-based computational methods have been proposed [9–12]. gkmSVM (gapped K-mer support vector machine) is a machine learning algorithm developed by [9]. The gkmSVM algorithm takes advantage of gapped k-mers, which are short DNA sequence patterns with gaps, to capture important motifs and patterns in the DNA sequence [9], for example underlying a genomic feature, such as a ChIP-seq peak. Its ability to handle gapped k-mers and find intricate sequence patterns makes it valuable in deciphering the regulatory code of the genome. It has been used in genomics research to prioritize and interpret the functional effects of genetic variants [13, 14]. Although gkmSVM is trained to distinguish sequences under peaks from other sequences, it can be used to predict the effect of a polymorphism on peak height and hence the magnitude of ASB. It does this by comparing the probability that the two allelic sequences occur under a peak.

gkmSVM offers an advantage by predicting asbQTL even in the absence of ASB data because it compares sequences beneath ChIP-seq peaks with other sequences. However, in cases where ASB data exists, we should be able to use this information to predict asbQTL and distinguish them from those non-causal ASB. We, therefore, developed a novel BLUP (best linear unbiased prediction) model, to predict ASB at heterozygous sites using the flanking DNA sequences and phenotype of ASB variants.

Genome regions with histone modification are likely to include enhancers and promotors of gene expression. Consequently, variants that affect the height of the ChIP-seq peak may also affect gene expression. This study has two main aims: (1) predict potential causal variants responsible for ASB at ChIP-seq peaks (i.e., asbQTL), and (2) investigate whether these candidate asbQTL play a causal role in gene expression variation within the mammary gland of lactating cows. The novel analysis uses DNA sequences that contain the same heterozygous site and occur in multiple places in the genome. If it is the same allele at the heterozygous site that is usually associated with the higher ChIP-seq peak, this implies that the heterozygous site is causing the difference in peak height.

## Methods

### ChIP-seq peaks and ASB of ChIP-seq peaks

**Fig. 1:**
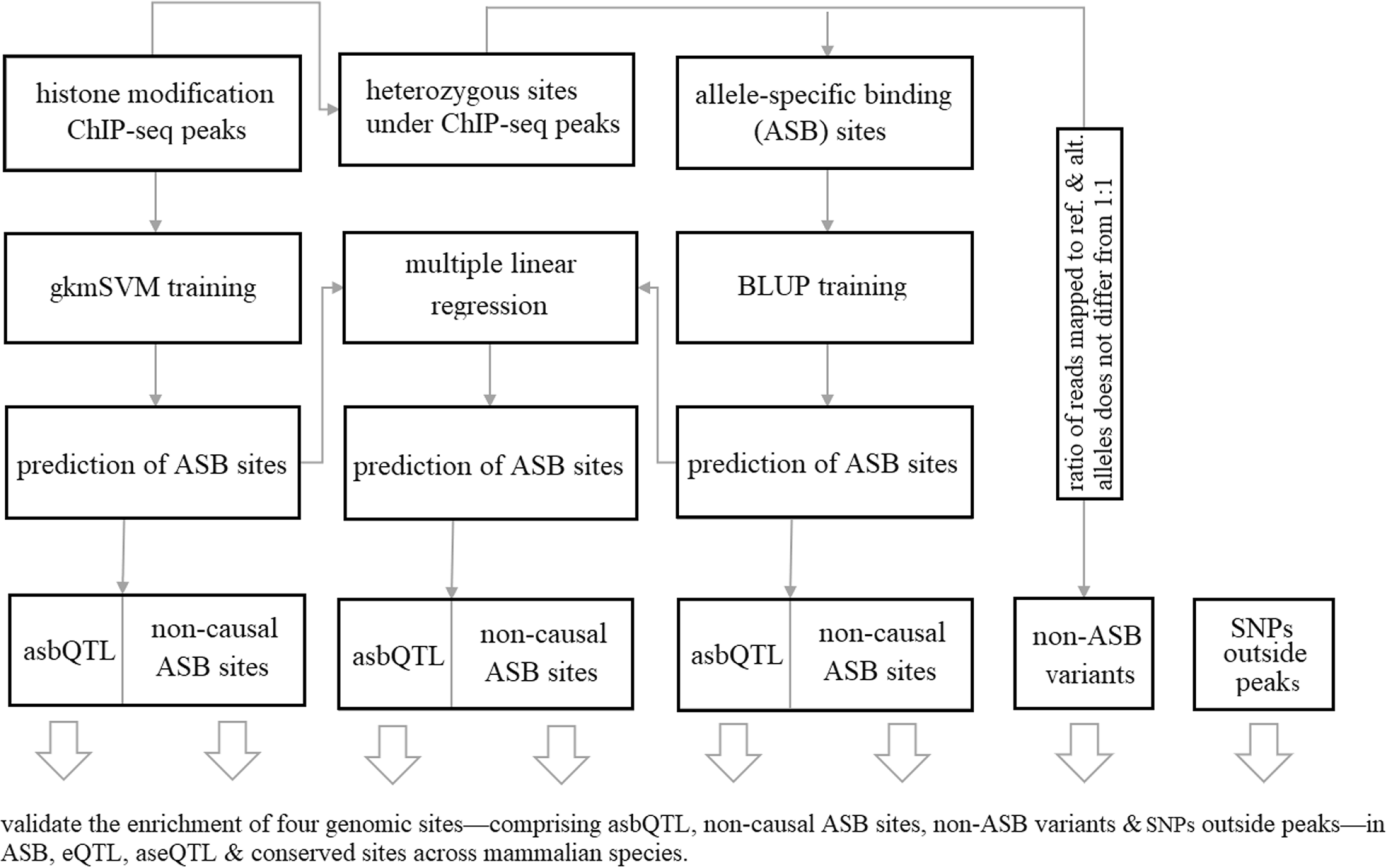
Schematic overview of the study

The ChIP-seq peak data utilized in this study originates from an experiment conducted on mammary gland samples from 98 lactating cows, assayed for histone modifications H3K4me3 (trimethylated Histone3 Lysine4), and 37 of these cows were also assayed for H3K27ac (acetylated Histone3 Lysine27) (Table 1). This data was described by [7]. Raw fastq data are publicly available at https://www.ebi.ac.uk/ena/browser/view/PRJEB52456. Peaks were called by [7], in reference to the ARS-UCD1.2 [15] bovine genome. Figure 1 depicts a schematic overview of this study.

**Table 1:** Summary of the ChIP-seq peaks and allele-specific binding (ASB) data for histone modifications H3K4me3 and H3K27ac

Data	H3K4me3	H3K27ac
ChIP-seq peaks		
Number of animals	98	37
Number of consensus peaks^a^	10,211	9469
Number of Excluded peaks^b^	3740	2836
Remaining consensus peaks^c^	6471	6633
Random DNA sequence^d^	32,211	32,841
Allele-Specific Binding sites		
Total number of animals	98	37
Total number of SNPs under peak^e^	974,837	1,748,824
ASB sites (with χ^2^ *P* < 0.05)	492,370	630,726
ASB in at least 5% of cows	66,121	61,881
SNPs removed (tri-allelic)	9	0
ASB sites used for the analyses	66,112	61,881

^a^Peaks that appeared in at least 50% of the biological samples; ^b^Peaks located on non-autosomes or were of length > 2000 bp; ^c^Positive sequences used to train gkmSVM; ^d^Negative sequences used to train gkmSVM; ^e^ASB and non-ASB sites under peak

**Fig. 2:**
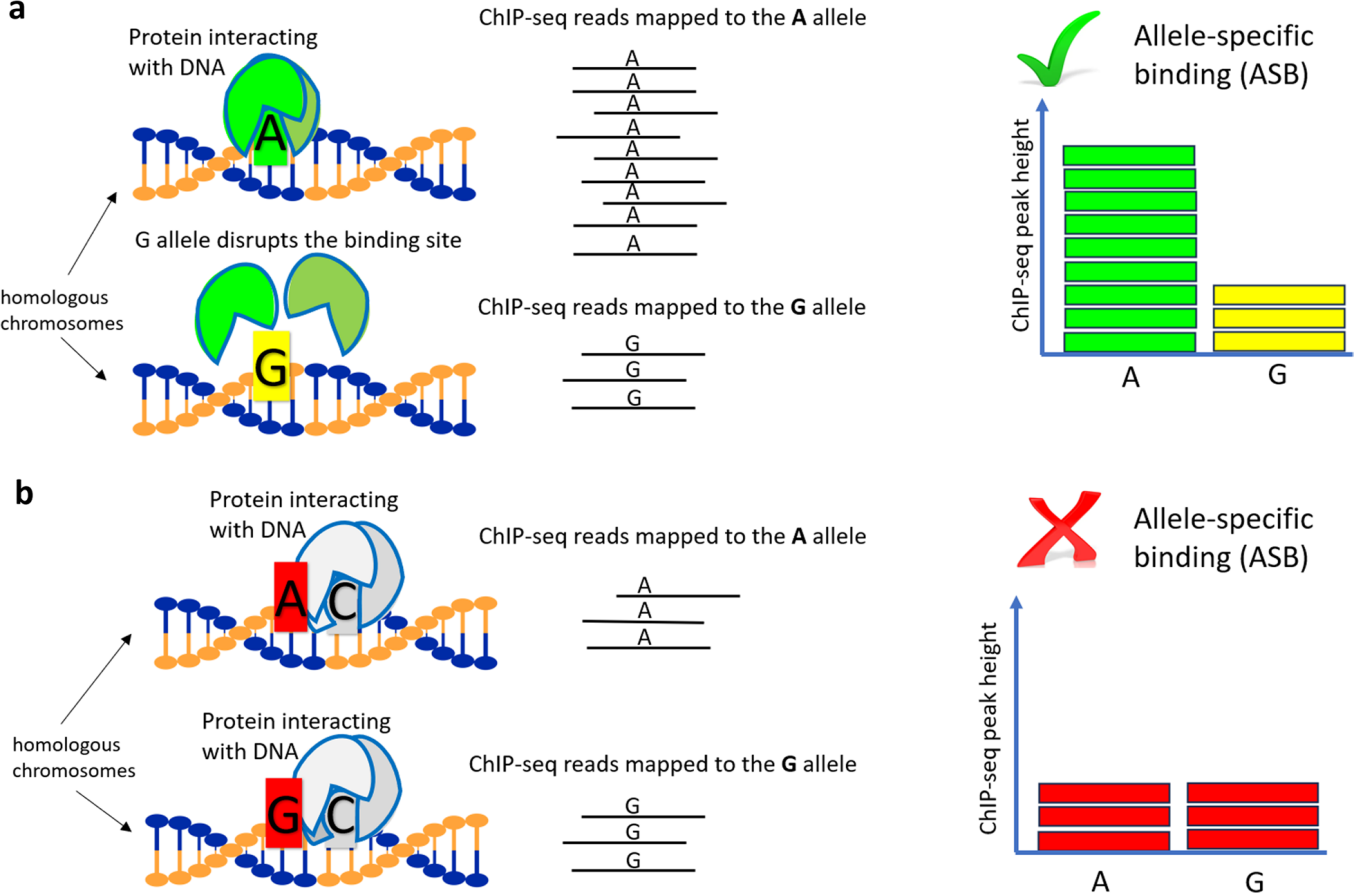
Overview of **a** allele-specific binding (ASB) and **b** non-ASB phenomena within a ChIP-seq peak region

In addition, we used ASB data that are heterozygous sites under ChIP-seq peaks [7] where the number of ChIP-seq reads mapped to either reference or alternate allele is significantly different from the numbers mapped to the other allele (Fig. 2). Reference and alternate alleles could only be defined if there were heterozygous sites in the ChIP-seq peak [7]. Summary statistics including number of peaks and SNPs under peaks per individual are presented in Additional file 1.

Heterozygous sites under ChIP-seq peaks were removed from the data if (see Table 1):

1. The difference between the number of reads mapped to reference and alternate alleles was insignificant (i.e., non-ASB variants; *P* > 0.05). The *P* value was based on the statistic $x=\frac{{(ref-alt)}^{2}}{N}$, in which $x$ follows a χ^2^ distribution with 1 degree of freedom; *N* is the total number of reads; and ref & alt represent the number of reads mapped to reference & alternate alleles.
2. They showed ASB but appeared in less than 5% of cows.
3. They were not biallelic SNPs.

We then aggregated ASB events per site, by summing up the number of ASB reads across cows. This procedure resulted in a new dataset with only one ASB record (aggregated) per site, where the read counts for the reference and alternate alleles now representing the total counts across cows. Therefore, we defined a phenotype for each ASB site as follows: y = $\frac{alt}{N}-0.5$, where the phenotype (y) represents the observed difference between ChIP-seq peak height on homologous chromosomes (i.e., the magnitude of ASB), the sign of y indicates the direction of ASB and the -0.5 results in y = 0 if there is no ASB.

These ASB data were used to train a predictor of ASB from the DNA sequence surrounding the heterozygous sites (i.e., BLUP model).

### BLUP training and prediction of ASB

Aggregated phenotypes from 66,112 H3K4me3 ASB sites and 61,881 H3K27ac ASB sites were used separately (Table 1) to train a prediction of ASB using the 5 bp on either side of the heterozygous site. The surrounding DNA sequences of 11 bp (i.e., five bp from either side with the variant allele in the middle) were extracted for all the ASB sites from ARS-UCD1.2 [15], using *genNullSeqs* function [16].

The following steps were conducted to predict variant impact for a given ASB (target) site:

1. For each target ASB site, we generated a training set consisting of all other ASB sites that had the same genotype as the target site and the flanking 10 bases matched, in at least seven positions, to the flanking 10 bases of the target site.
2. Now, we have a target ASB site and a subset of other ASB sites as a training set. For training sites, the phenotype is modelled as ${\mathbf{y}}_{\mathbf{t}\mathbf{r}\mathbf{a}\mathbf{i}\mathbf{n}}=\boldsymbol{1}\text{u}+\mathbf{e}$, where $\text{u}$ is the average phenotype across the training set, and ${\mathbf{y}}_{\mathbf{t}\mathbf{r}\mathbf{a}\mathbf{i}\mathbf{n}}$ and $\mathbf{e}$ are vectors containing phenotypes and residuals for the training sites, respectively. Therefore, $\mathbf{R}=\mathbf{I}( \frac{1}{4\text{N}}+{\upsigma }_{\text{s}}^{2})$, and ${\mathbf{R}}^{-1}=\mathbf{I}\frac{4\text{N}}{4\text{N}{\upsigma }_{\text{s}}^{2}+1}$, where $\mathbf{R}$ represents var (**e**), $\mathbf{I}$ is the identity matrix, $\text{N}$ is the total number of reads for each training ASB site, and ${\upsigma }_{\text{s}}^{2}$ represents variance of ${\mathbf{y}}_{\mathbf{t}\mathbf{r}\mathbf{a}\mathbf{i}\mathbf{n}}$ that is not explained by binomial sampling of N sites.
3. To predict the target ASB phenotype, we can now use the BLUP equation $\widehat{\text{u}}=\frac{\sum {\mathbf{R}}^{-1}\mathbf{y}}{\sum {\mathbf{R}}^{-1}+ {\upsigma }_{\text{u}}^{-2}}$, where $\widehat{\text{u}}$ is the predicted phenotype for the target ASB site and ${\upsigma }_{\text{u}}^{-2}$ is the inverse of var (u).
4. We used a diverse range of grid values for ${\upsigma }_{\text{s}}^{2}$ and ${\upsigma }_{\text{u}}^{2}$ to find the best values that maximizes the correlation between ${\mathbf{y}}_{\mathbf{t}\mathbf{a}\mathbf{r}\mathbf{g}\mathbf{e}\mathbf{t}}$ (observed phenotype for ASB) and $\widehat{\mathbf{u}}$ (predicted phenotype for ASB using the other ASB sites).

### gkmSVM training with ChIP-seq peak data

On average, we had ~ 104,000 H3K4me3 peaks and ~ 303,000 H3K27ac peaks per cow (see Additional file 1). The gkmSVM is trained using labelled sets of sequence data, where positive and negative training sets correspond to functional (e.g., ChIP-seq peaks) and non-functional (random) DNA sequences, respectively. Therefore, training data for gkmSVM was prepared as follows:

1. only peaks with − Log10 *P* value > 200 obtained by MACS2 [17] were retained. The logic behind this is that classification performance of gkmSVM was low when applying less stringent *P* value.
2. we extracted peaks that appeared in at least 50% of the biological samples through consensus voting [18].
3. peaks located on non-autosomes or > 2000 bp in length were excluded from downstream analyses. These steps retained 6471 H3K4me3 peaks and 6633 H3K27ac peaks, labelled as positive sequences required for training gkmSVM models (Table 1).
4. The *genNullSeqs* function from the R gkmSVM package [16] was used to extract a five times larger set of equally sized and GC content matched sequences from ARS-UCD1.2 [15] for the negative training set (Table 1).

A kernel matrix was then computed using the *gkmsvm_kernel* function with positive and negative training sets as input. Subsequently, the *gksvm_train* function was used to train gkmSVM models for the two histone modifications. This resulted in a prediction or weight for every 10 bp sequence, which represents the probability of it being under a peak.

To evaluate the classification performance of gkmSVM, a five-fold cross validation was performed, where 20% of the training data was masked each time to test the model trained on the other 80%.

### Prediction of ASB using the trained gkmSVM

For a given ASB site, we extracted the 19 bp length (i.e., $L\times 2-1$; here L = 10 refers to the sequence length used for training gkmSVM) flanking sequences for each ASB site (Table 1) from ARS-UCD1.2 [15], masked the middle position, and duplicated the masked sequence. The masked position of one duplicate was filled with the reference allele, while the other was filled with the alternate allele, enabling exploration of the effect of variant alteration in the regions of interest. Therefore, we had two lists of 19 bp length sequences that only differed by one base in the middle position. Then the *gkmsvm_delta* function was used to calculate deltaSVM [13] as follows:

$$\text{deltaSVM}= \sum_{\text{i}=1}^{\text{s}=10}\left[{\text{w}}_{\text{i }(\text{alt})}-{\text{w}}_{\text{i }(\text{ref})}\right],$$

where $s$ represents sliding windows of 10 bp length (step size = 1) in the 19 bp paired sequences that contain reference and alternate alleles, and ${{w}_{i}}_{alt}$ and ${{w}_{i}}_{ref}$ represent SVM weights predicted for the corresponding ${i}^{th}$ sliding windows.

### Multiple linear regression

We fitted a multiple linear regression (MLR) model by combining the predictions of gkmSVM and BLUP as follows:

$$\mathbf{y}={\boldsymbol{1}\upbeta }_{0}+{\upbeta }_{1}\widehat{\mathbf{u}} + {\upbeta }_{2}\widehat{\mathbf{d}\mathbf{e}\mathbf{l}\mathbf{t}\mathbf{a}\mathbf{S}\mathbf{V}\mathbf{M}}+\mathbf{e},$$

where $\mathbf{y}$ is a vector of response variables (i.e., ASB phenotypes), $\boldsymbol{1}$ is a vector of ones, $\widehat{\mathbf{u}}$ (from BLUP) and $\widehat{\mathbf{d}\mathbf{e}\mathbf{l}\mathbf{t}\mathbf{a}\mathbf{S}\mathbf{V}\mathbf{M}}$ (from gkmSVM) are predictor variables, and ${\upbeta }_{1}$ and ${\upbeta }_{2}$ are regression coefficients of the $\mathbf{y}$ on predictors, ${\upbeta }_{0}$ is the intercept term, and $\mathbf{e}$ is the residual.

### Identifying candidates asbQTL and assessing prediction accuracy

Once the impact of the ASB variants on the histone modifications were predicted, a subset of 1000 ASB sites, based on the magnitude of the predicted scores, were considered as candidate asbQTL for each of the three methods used. The accuracy of prediction on the direction of the candidate asbQTL was calculated. The accuracy is defined as the proportion of SNPs in which the predicted ASB direction matches the observed direction i.e., if it is negative, the count of the reference allele is higher than that of the alternate allele.

### Validation of the candidate asbQTL using independent data

To validate our result, we used independent ASB data, from a study of mammary tissue of 3 lactating Australian Holstein cows (validation data) [19]. In this dataset, a heterozygous site under a peak was considered allele-specific binding (ASB) if a significant difference was observed (*P* < 0.01) in allelic ratio of the reads mapped to the site.

Then, the proportion of ASB (validation) in each of the four different groups of genomic sites (listed below) was compared to that in the next group.

1. The 1000 sites with the highest magnitude of predicted ASB from each of our methods of identifying asbQTL from sequence.
2. non-causal ASB (i.e., ASB sites that were NOT predicted as asbQTL).
3. sites under a peak that were not ASB.
4. sites that were not under a peak.

We used a χ^2^ test and considered results with *P* < 0.01 as statistically significant.

### Do the candidate asbQTL affect gene expression?

We investigated if SNPs from the four categories of genomic sites were enriched in different functional annotations, including eQTL, aseQTL, and evolutionary conserved sites across mammalian species. eQTL (*cis*) were taken from CattleGTEx [20, 21] where they were significant across 16 tissues. aseQTL from blood and mammary gland [19] where variants with *P* < 5E−6 in any tissues were used. The choice of this *P* value threshold is based on experiences in single-tissue mapping CattleGTEx data [20]. Also, conserved sites including (1) those conserved across 30 mammals (obtained from http://hgdownload.cse.ucsc.edu/goldenpath/hg38/phastCons30way); and (2) those conserved across 100 vertebrates (obtained from http://hgdownload.cse.ucsc.edu/goldenpath/hg38/phastCons100way) were used. The definition of being conserved was a PhastCons score > 0.9 as described in [22]. Finally, the proportion of eQTL, aseQTL, and Conserved sites in each of the four different groups of genomic sites (listed above) were compared to that in the next group.

## Results

### Optimizing BLUP model parameters to predict causal ASB sites

To predict ASB variants using the BLUP model, we tried a diverse range of grid values for ${\upsigma }_{\text{u}}^{2}$ and ${\upsigma }_{\text{s}}^{2}$ to find the best predictions that maximized the correlation between $\mathbf{y}$ and $\widehat{\mathbf{u}}$. The correlations for H3K4me3 ranged from + 0.118 to + 0.192, and for H3K27ac ranged from + 0.120 to + 0.151. The highest correlation for H3K4me3 was obtained with ${\upsigma }_{\text{u}}^{2}=0.001$ and ${\upsigma }_{\text{s}}^{2}=0.035$, and for H3K27ac with ${\upsigma }_{\text{u}}^{2}=0.003$ and ${\upsigma }_{\text{s}}^{2}=0.07$. Therefore, we used these values to predict ASB with the BLUP model.

### Ability of gkmSVM to predict sequences under ChIP-seq peaks

**Fig. 3:**
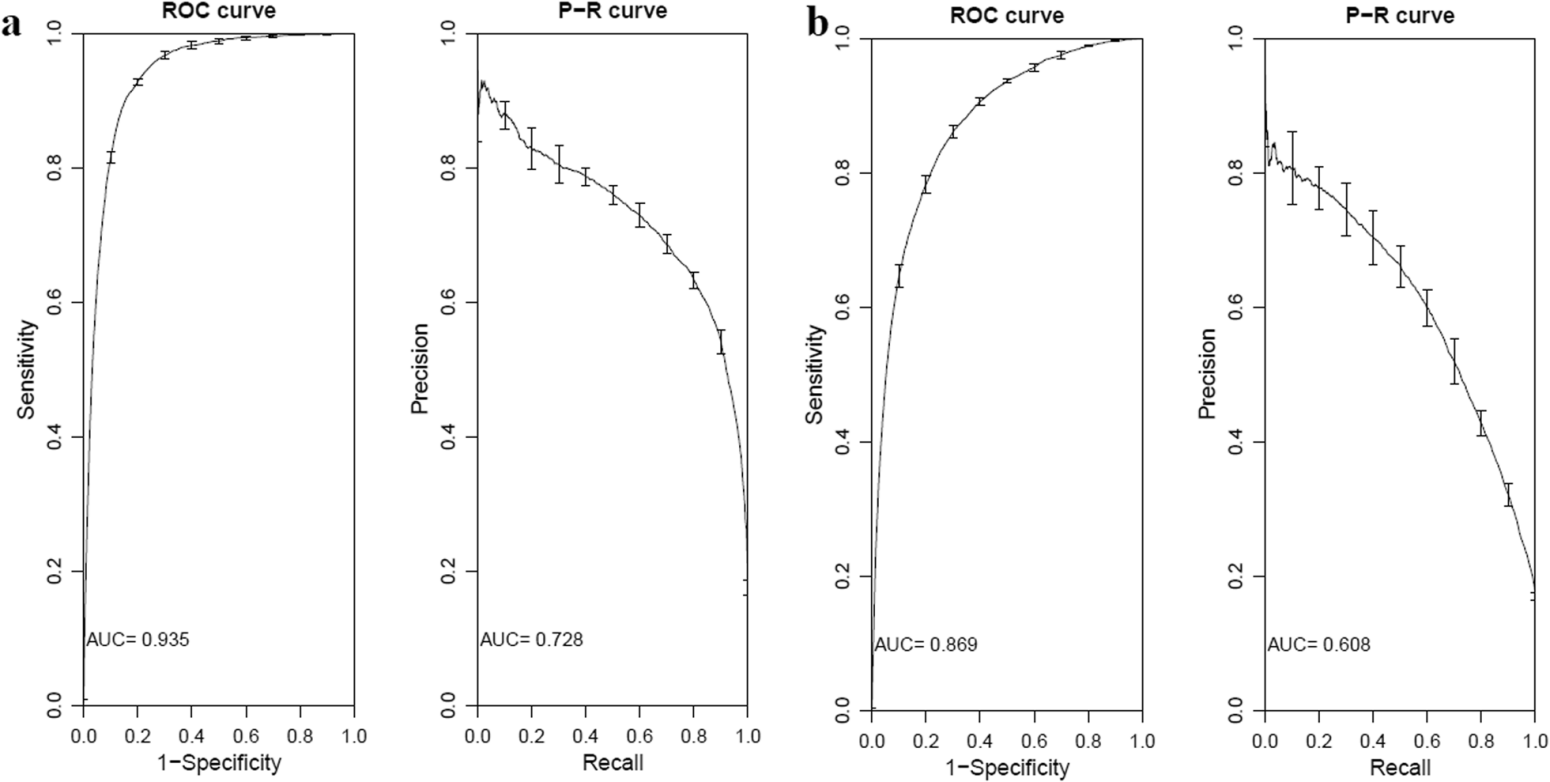
auROC and auPRC representing five-fold cross validation using gkmSVM training on histone modifications **a** H3K4me3 and **b** H3K27ac

As shown in Table 1, about 6.5K peaks (positive sequences) and a 5 times larger set of negative sequences were used for gkmSVM training of each histone modification. The performance of gkmSVM five-fold cross validation with training data is shown in Fig. 3. The area under curve (auROC) and precision recall curve (auPRC) for H3K4me3 was 0.935 and 0.728, and for H3K27ac 0.869 and 0.608, respectively. This shows that gkmSVM could discriminate sequences that are under peaks from those that are not.

### Prediction accuracy for the candidate asbQTL set

**Fig. 4:**
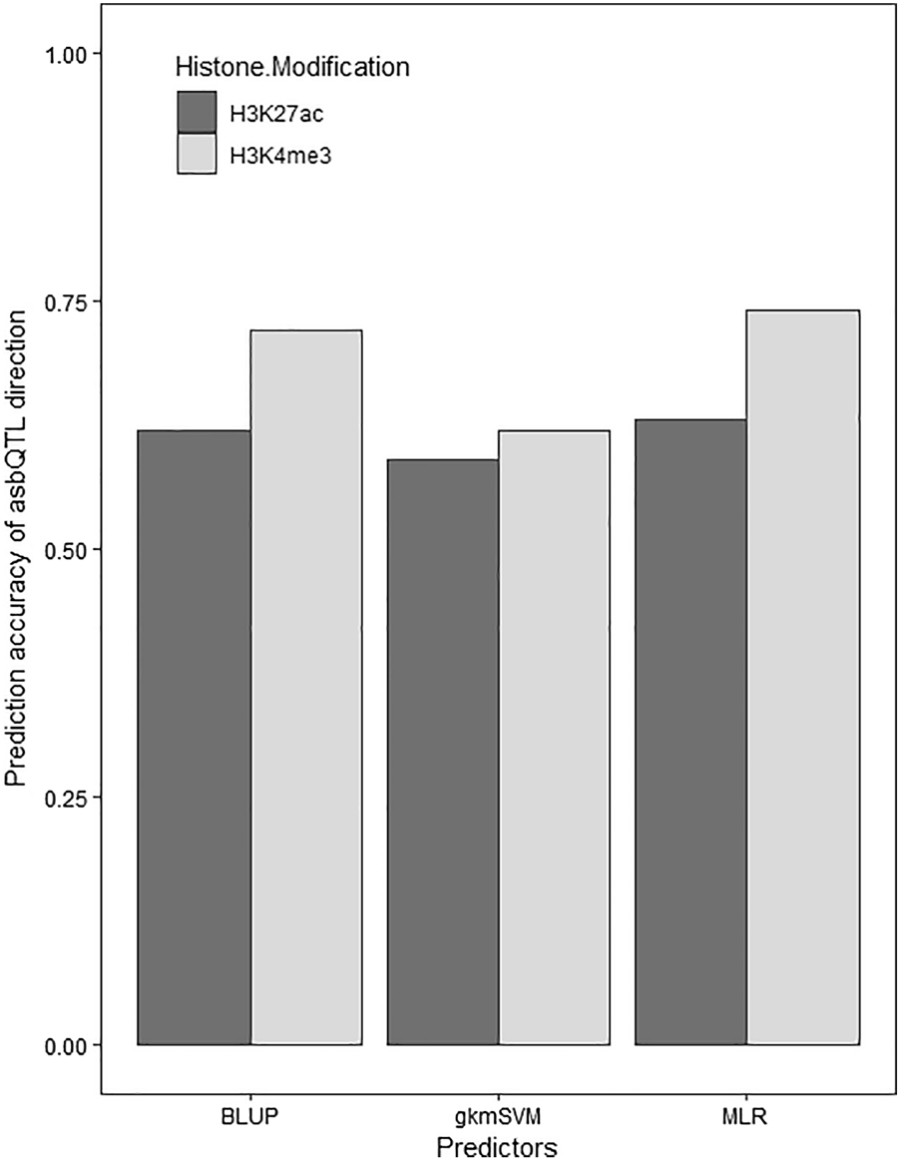
Accuracy for candidate asbQTL variants predicted by gkmSVM, BLUP and multiple linear regression (MLR) for the two histone modifications

The ability of the three methods to predict the direction of ASB in the candidate asbQTL set from the two histone modifications (H3K4me3 and H3K27ac) is shown in Fig. 4. Accuracy of prediction for H3K4me3 with gkmSVM, BLUP and MLR was 0.62, 0.72, and 0.74, respectively. In addition, prediction accuracy for H3K27ac was 0.59, 0.62 and 0.63 for the same models, respectively. This result suggests lower predictability for H3K27ac compared to H3K4me3, confirmed by both gkmSVM and BLUP models. Prediction accuracy of BLUP for histone modification H3K4me3 was significantly higher than that for gkmSVM (*P* < 0.01), but the difference was not significant for H3K27ac (*P* > 0.05). The impact of ASB variants on the histone modifications predicted with the three models are presented in Additional files 2 and 3.

### Multiple linear regression (MLR)

Prediction accuracy of the MLR model for the two histone modifications is shown in Fig. 4. The MLR model was fitted using both gkmSVM and BLUP scores as predictors to increase the power of prediction. Details of the fitted models are presented in Table 2. Regression coefficients of both predictors on the phenotype of ASB variants were significant, however, the adjusted R^2^ of the models were 0.039 and 0.024 for H3K4me3 and H3K27ac, respectively. This result suggests that despite the statistically significant effect of both predictors on ASB phenotypes (response variable), only a small proportion of the variance of ASB is explained by the predictors.

**Table 2:** Multiple linear regression (MLR) model using BLUP and gkmSVM as predictor variables for two histone modifications

Coefficient	H3K4me3		H3K27ac	
	Estimate (SE)	*P* value	Estimate (SE)	*P* value
Intercept	− 0.003 (0.0005)	9E−10	− 0.006 (0.0007)	2E−16
BLUP	1.274 (0.02)	2E−16	0.980 (0.026)	2E−16
gkmSVM	0.010 (0.0009)	2E−16	0.008 (0.001)	6E−12

The adjusted R^2^ for the H3K4me3 model was 0.039 and for the H3K27ac was 0.024

### Functional annotation of the candidate asbQTL set and the other genomic sites

Table 3 presents the proportion of the candidate H3K4me3 asbQTL set and the 3 other categories of genomic sites that were annotated as ASB, eQTL, aseQTL, and conserved sites in the validation data. For instance, of the 1000 sites classified by our BLUP method as asbQTL, 16.9% were confirmed in independent data, compared to 13.5% of other ASB sites, 2.3% of sites under ChIP-seq peaks that are not ASB and 0.2% of sites not under ChIP-seq peaks. (All these differences are significant at *P* < 0.01). This result suggests that we have, to some extent, successfully identified genetic variants causing changes in allele specific binding in H3K4me3 histone modification peaks.

**Table 3:** The proportion of functionally annotated (H3K4me3) variants within each category, including candidate asbQTL, non-causal ASB sites, non-ASB variants under peak and SNPs from outside peaks

Functional annotation	Candidate asbQTL^a^			non-causal ASB sites (N = 59,682)	Non-ASB variants under peak (N = 44,021)	SNPs not under peak (N = 37,801)
	MLR	gkmSVM	BLUP			
ASB	0.161	0.154	0.169*	0.135*	0.023*	0.002
eQTL	0.522	0.531	0.522	0.637*	0.334*	0.157
aseQTL	0.507	0.503	0.507	0.560*	0.295*	0.171
Conserved sites	0.08	0.104	0.08	0.117*	0.085*	0.071

^a^The top 1000 ASB variants based on the magnitude of the predicted scores by each method were considered as candidate asbQTL

An asterisk (*) indicates statistical significance (*P* < 0.01) when the values in the current column are statistically greater than those in the next column

However, the asbQTL showed no further enrichment for eQTL/aseQTL or conserved sites compared to the other ASB sites under a peak (*P* > 0.05). This result suggests that causal variants affecting asbQTL do not affect gene expression. We also found that ASB sites compared to non-ASB sites, and the latter compared to the random SNPs outside peak, are enriched in eQTL/aseQTL (Table 3).

For histone modification H3K27ac, there were no significant differences in any of the annotations between the four categories of genomic sites (Table 4). This can be attributed to the poorer predictability of sequences causing histone modification H3K27ac peaks as shown in Fig. 4.

**Table 4:** The proportion of functionally annotated (H3K27ac) variants within each category, including candidate asbQTL, and non-causal ASB sites

Functional annotation	Candidate asbQTL^a^			non-causal ASB sites (N = 57,802)
	MLR	gkmSVM	BLUP	
ASB	0.119	0.135	0.122	0.128
eQTL	0.544	0.507	0.522	0.578
aseQTL	0.48	0.471	0.46	0.525
Conserved sites	0.093	0.084	0.088	0.09

^a^The top 1000 ASB variants based on the magnitude of the predicted scores by each method were considered as candidate asbQTL. Candidate asbQTL for the histone modification H3K27ac did not show enrichment for ASB, nor did they for the other functional annotations, compared to the non-causal ASB sites

## Discussion

Mutations occurring within functional regions, such as enhancers or promoters, have the potential to disrupt the regulation of gene expression and consequently affect the phenotype of complex traits. These mutations can be identified by assays designed to detect functional regions in DNA such as ChIP-seq peaks [7], which were used in this study. However, genomic sites may be associated with complex traits, gene regulation, or functional assays because they are in LD with the site causing the change in phenotype, making the search for causal variants more difficult [7, 10, 13]. The primary objective of this research was to use sequence-based computational methods to pinpoint SNPs causing variation in the level of allele specific binding in histone modification peaks (i.e., asbQTL) in the mammary gland of dairy cattle while distinguishing these causal variants from non-causal variants associated with variation in peak height due to LD.

If histone modifications mark sites of TF binding, we expect that there will be some similarity between the sequences underlying these histone modifications, and therefore, the possibility to predict which sequences underly ChIP-seq peaks marking histone modifications. gkmSVM [9] has been designed to do this by comparing sequences under functional marks with other sequences. In most cases gkmSVM has been used to predict sequences under DNase hypersensitivity regions [13, 14, 23], but it was also used to predict sequences under histone modification ChIP-seq peaks [10]. We determined how well gkmSVM and our new method (an adaption of BLUP) could predict the effect of an allelic difference on the height of the ChIP-seq peak.

In the search for causal mutations underlying peak height, evidence of causality unaffected by LD would be greatly beneficial [7, 10, 13]. A heterozygous site which is under a ChIP-seq peak can show ASB for one of two reasons—it causes the ASB, or it is in LD with the causal site. If the direction of ASB can be predicted from the surrounding sequence, this is strong evidence that the site causes the ASB because there is no reason that the sequence surrounding a linked site would be consistent. gkmSVM does not need data on ASB sites to train its prediction of ASB. However, when data on ASB exists it can be used to train a predictive model. Hence, we developed a novel BLUP model which exploits the phenotype of ASB sites within ChIP-seq peaks as well as their flanking DNA sequences to predict mutations affecting peak height. We hypothesized that if multiple ASB sites with the same SNP genotype (e.g., AG) share similar DNA sequences, and the ASB direction is consistent across these sites (e.g., A > G), then they might be causal mutations affecting ChIP-seq peak height, independent of LD, because they are consistent across multiple sites. In other words, causal sites with similar sequences are expected to show consistent ASB direction, whereas sites that show ASB due to LD may not share similar sequences or have consistent direction of ASB. Therefore, the BLUP model predicts an ASB site based on the other ASB sites, and their flanking DNA sequences. Alternatively, gkmSVM weights kmers based on their enrichment under peaks compared to the random DNA sequences. These two methods have the same objective with different strategies and inputs.

We validated our result by comparing putatively causal ASB (i.e., asbQTL) and non-causal ASB in independent data. That is, ASB from independent data was significantly more frequent (*P* < 0.01) in the predicted asbQTL than in the non-causal ASB variants. Therefore, we were, to some extent, successful in identifying functional variants causing peak height of H3K4me3 because predicting ASB at the target site based on data from other sites was successful. Both gkmSVM and the BLUP method were successful, and they were correlated to a small extent. However, combining the two methods via a multiple regression gave accuracies very little higher than the BLUP method.

If the asbQTL is also causal for gene expression, then it should show enrichment for eQTL/aseQTL. Our results show that eQTL/aseQTL (eQTL from 16 different tissues; see Methods) are more common under ChIP-seq peaks than elsewhere and more common at sites that show ASB than at sites that do not show ASB but are under ChIP-seq peaks. However, eQTL/aseQTL (eQTL from 16 different tissues; see Methods) were not more prevalent at the predicted causal ASB sites (i.e., asbQTL) compared to the non-causal ASB sites. The same result was also obtained when only using mammary gland cis eQTL data (results not shown). This last result suggests the alternative explanation, that is, sites that cause ASB are not often causal sites for eQTL/aseQTL. For instance, presence of a specific TF can impact the height of multiple peaks, but most of these peaks do not impact gene expression.

If the height of a peak correlates positively across tissues with expression of a gene, this might suggest that a SNP that alters the height of the peak would affect the expression of the linked gene. Prowse-Wilkins et al. [24] found that even when the height of a peak was positively correlated across 22 tissues with expression of a gene, ASB of the peak couldn’t predict ASE of the gene. This result suggests that the correlations between peak height and gene expression do not always indicate that the height of the peak causes the extent of gene expression. Our result and those of [24] could both reflect the same underlying biology that height of the histone modification peak does not directly drive expression of the gene it is correlated with. However, our study differs from those of [24], that is, they knew which exon-peak pairs to look at but didn’t care about which ASB site is causal. Contrarily, we predicted putatively causal ASB sites and tested if they do affect any of the nearby genes.

It is possible that our result that ASB sites do not necessarily cause ASE, is influenced by a lack of statistical power due to LD. Indeed, non-causal sites which show ASB must be in LD with sites that do cause ASB. Consequently, if the causal site is also an eQTL/aseQTL then the non-causal site will also be associated with gene expression to some extent. That is, we may lack power to distinguish between our putatively causal ASB sites and other non-causal ASB sites with respect to gene expression.

It has been reported that DNase-seq (DNase I Hypersensitive sites sequencing) or DNase-seq flanked by histone modification ChIP-seq are more predictive of gene expression than histone modifications alone. For instance [13] trained a gkmSVM model using DNase-seq data on human lymphoblastoid GM12878 cell lines. The trained model could moderately discriminate 574 highly confident dsQTL (SNPs associated with DNase hypersensitivity) versus a 50 times larger set of non dsQTL SNPs (auROC = 0.75; auPRC = 0.19). Beer [23] reported that training gkmSVM on DNase-seq peaks flanked by histone modification peaks for H3K27ac or H3K4me1 in human K562 cell lines is more predictive of MPRA (Massively Parallel Reporter Assays) expression (auROC = 0.83) than DNase-seq alone (auROC = 0.79). One of the limitations of the current study in cattle is that we do not have any DNase-seq data, or an equivalent assay. The variation seen between histone modification and DNase-seq could possibly be attributed to a combination of biological and technical factors. The biological process measured by these assays vary. DNase-seq peaks mark open chromatin, indicating the presence of proteins interacting with the DNA in a relatively narrow region of ~ 300–500 bp. Therefore, the DNase signal is primarily influenced by the nearby DNA sequence, particularly the binding of TFs. Histone modification ChIP-seq provides a more intricate assessment of chromatin state, indicating the presence of H3K4me3 at active promoters, H3K27ac at active promoters and enhancers, and H3K4me1 at active enhancers [10]. Sequence-based prediction of histone modification can therefore be less accurate due to spatial histone modification spreading over a broader region of chromatin, the influence of trans-factors, and the greater width of these marks [10], sometimes up to 2000 bp. Another possible reason is that many variants that change histone modification are regulatory dead ends. This means they do not result in a change in gene expression, at least at the time point or tissue they are being measured in. Regulation of gene expression is time and cell type dependent, so the effect of a single genetic variant is not constant.

Genomic sites under ChIP-seq peaks, including ASB sites and putatively causal ASB sites, are only slightly enriched for sites that are conserved across mammals (Table 3). This implies that these regulatory regions are not greatly constrained in evolution.

## Conclusions

This research aimed to pinpoint SNPs responsible for variation in allele specific binding at histone modification peaks and explore whether these variants are also causal in regulating gene expression. To achieve this, we developed a novel BLUP model and used gkmSVM to predict causal mutations. We showed that the surrounding sequence could identify variants causing ASB at H3K4me3 peaks. However, intriguingly, the candidate asbQTL variants did not exhibit significantly greater enrichment for eQTL/aseQTL compared to the other ASB sites under a peak. This suggests that many sites influencing histone modification peak height may not directly impact gene expression. Nevertheless, it is important to acknowledge that our ability to distinguish between causal and non-causal ASB sites that are in LD with causal sites regarding their impact on gene expression, may be limited by statistical power. In conclusion, this research highlights the challenges involved in identifying causal variants underlying histone modification peak height and their relationship with gene expression. Integrating additional datasets such as DNase-seq with histone modification may enhance our understanding of the functional implications of genetic variants on gene regulation mechanisms.

### Supplementary Information

**Additional file 1: Table S1.** Summary statistics of the initial number of peaks and number of SNPs under peaks per individual for the two histone modifications H3K4me3 and H3K27ac.

**Additional file 2: Table S2.** Variant impact scores for ASB sites under histone modification H3K4me3 ChIP-seq peaks.

**Additional file 3: Table S3.** Variant impact scores for ASB sites under histone modification H3K27ac ChIP-seq peaks.

## Data Availability

ChIP-seq data is available here: https://www.ebi.ac.uk/ena/browser/view/PRJEB52456.
